# Effect of Biomass Drying Protocols on Bioactive Compounds and Antioxidant and Enzymatic Activities of Red Macroalga *Kappaphycus alvarezii*

**DOI:** 10.3390/mps7060088

**Published:** 2024-11-01

**Authors:** Aline Nunes, Felipe de Souza Dutra, Sinara de Nazaré Santana Brito, Milene Stefani Pereira-Vasques, Gadiel Zilto Azevedo, Alex Ricardo Schneider, Eva Regina Oliveira, Alex Alves dos Santos, Marcelo Maraschin, Fábio Vianello, Giuseppina Pace Pereira Lima

**Affiliations:** 1 Plant Biotechnology and Postharvest Laboratory, Department of Chemical and Biological Sciences, Institute of Biosciences, São Paulo State University, Botucatu 18618-970, SP, Brazil; sinara.santana@unesp.br (S.d.N.S.B.); milene.pereira@unesp.br (M.S.P.-V.); pace.lima@unesp.br (G.P.P.L.); 2 Laboratory of Biotechnology of Natural and Synthetics Products, Institute of Biotecnology, University of Caxias do Sul, Caxias do Sul 95070-560, RS, Brazil; fsdutra@ucs.br (F.d.S.D.); arschneider1@ucs.br (A.R.S.); 3 Laboratory of Plant Morphogenesis and Biochemistry, Department of Plant Science, Federal University of Santa Catarina, Florianópolis 88034-000, SC, Brazil; gadiel.azevedo@grad.ufsc.br (G.Z.A.); regina.oliveira@posgrad.ufsc.br (E.R.O.); m2@cca.ufsc.br (M.M.); 4 Aquaculture and Fisheries Development Center, Company of Agricultural Research and Rural Extension of Santa Catarina, Florianópolis 88010-970, SC, Brazil; alex@epagri.sc.gov.br; 5 Department of Comparative Biomedicine and Food Science, Università degli Studi di Padova, 35020 Legnaro, Italy; fabio.vianello@unipd.it

**Keywords:** freeze-dried, oven-dried, phenolic compounds, antioxidants, enzymes, seasonality

## Abstract

*Kappaphycus alvarezii* is a red seaweed used globally in various biotechnological processes. To ensure the content and stability of its bioactive compounds postharvest, suitable drying protocols must be adopted to provide high-quality raw materials for industrial use. This study aimed to analyze the influence of freeze-drying and oven-drying on the total phenolic content (TPC), total flavonoid content (TFC), antioxidant activity (FRAP and DPPH assays), total carotenoid content (TC), and lipase (LA) and protease activity (PA) of *K. alvarezii* samples collected over the seasons in sea farms in southern Brazil. The freeze-drying technique was found to be more effective regarding superior contents of TPC (39.23 to 127.74 mg GAE/100 g) and TC (10.27 to 75.33 μg/g), as well as DPPH (6.12 to 8.91 mg/100 g). In turn, oven-drying proved to be the best method regarding the TFC (4.99 to 12.29 mg QE/100 g) and PA (119.50 to 1485.09 U/g), with better performance in the FRAP (0.28 to 0.70 mmol/100 g). In this way, it appears that the drying process of the algal biomass can be selected depending on the required traits of the biomass for the intended industrial application. In terms of cost-effectiveness, drying the biomass using oven-drying can be considered appropriate.

## 1. Introduction

Seaweed has been used as food and medicine for at least 14,000 years [1]. However, large-scale cultivation is a relatively recent branch of aquaculture, dating back to the mid-20th century, when the global industrial production of algae was close to zero tons [2]. The increase in algae productivity worldwide has occurred especially in the last 20 years, with the global production of algal biomass, including both cultivation and wild collection, augmenting from 11.8 million tons to 35.8 million tons, i.e., ~3×, between 2000 and 2019 [3]. It is estimated that the global seaweed market could reach USD 24.9 billion in 2028, with a compound annual growth rate (CAGR) of 7.51% in the 2021–2028 period [4].

*Kappaphycus alvarezii*, the fifth most cultivated seaweed in the world, has been widely studied for its use in the food, pharmaceutical, and agricultural industries. Belonging to the Florideophyceae class and Rhodophyceae division, such a species can be found with red, yellow, brown, and green colors according to its contents of pigments and its nutrient status. Due to its fast growth (increase ~4.5% per day), differing from species that grow between 1 and 3% per day, this species is the main industrial source of κ-carrageenan, a polysaccharide representing up to 37% of the seaweed’s dry mass. Beyond the polysaccharide, it contains other carbohydrates, fatty acids, proteins, lipids, amino acids, and phenolic compounds [5,6]. Studies have demonstrated its antioxidant and anti-inflammatory effects in vitro [7], as well as its antitumor activity in vitro [8] and in vivo in rats [9]. Additionally, *K. alvarezii* has exhibited in vitro [10] and in vivo (mostly murine and *Macaccus rhesus*) antiviral properties [11] and has also shown promising antidiabetic activities in vitro [12]. Furthermore, the ethanolic extract of this macroalga has been investigated for its therapeutic potential in addressing neural dysfunction, with positive results found with in vitro study models [13].

However, a series of factors can interfere with the concentration of bioactive compounds in seaweed, which could directly impact its industrial applications requiring composition standardization. Climatic factors (especially exposure to light, temperature, salinity, and heat) during cultivation, as well as the cultivation location and biomass processing methods postharvest, along with inadequate storage, can lead to modifications in the composition and degradation of bioactive compounds [14]. For this reason, it is important to consider all these factors when analyzing the composition of seaweed.

In this context, the drying method adopted is crucial to prevent the degradation of molecules with nutraceutical properties. High-temperature drying, for instance, can degrade molecules, leading to the loss of important compounds such as vitamins, minerals, and heat-sensitive bioactive compounds like carotenoids and polyphenols [15,16].

Such an approach is particularly relevant for drying the biomass of aquatic products, which can contain up to 85% water, in order to minimize chemical and enzymatic reactions that lead to a reduction in nutrients, as well as stimulating microbial contamination [17]. Water molecules can interact with macromolecules through hydrogen bonding, disrupting their structure and function. By removing water, these interactions are minimized, allowing macromolecules to retain their native conformation. Additionally, water can promote the oxidation and degradation of bioactive compounds. In a dry environment, these reactions are significantly reduced, enhancing the stability of macromolecules. Furthermore, the removal of water can prevent microbial growth and enzymatic activity, which can further degrade bioactive compounds [18,19,20]. In this way, the maximization of biomolecule stability during biomass storage is possible through appropriate drying techniques, ensuring the quality of the raw material for industrial applications [17].

Among the commonly used drying techniques, freeze-drying and oven-drying can be found. These processes have different characteristics, especially in terms of cost, processing time, and efficacy in preserving the chemical composition of samples. Therefore, the choice between these techniques can be defined according to the final use of the algal biomass [18]. In this sense, this study aimed to investigate the effect of drying methods on the secondary metabolite profiles (e.g., phenolic, flavonoid, and carotenoid contents), antioxidant activity (DPPH and FRAP assays), and lipase and protease activities of *K. alvarezii* cultivated in two sites in southern Brazil, over the seasons of 2022. Therefore, the type of drying, seasonality, and place of cultivation are considered in this manuscript. Importantly, this is the first study to analyze the biomass of *K. alvarezii* produced in Brazil in this context.

By optimizing the drying process, we aim to enhance the quality of the raw material, thereby maximizing the biotechnological potential and applications of *K. alvarezii*, e.g., for use in agriculture, the main type of current marketing in the country, and for expansion into other areas, such as food and cosmetics. This research is a crucial step toward optimizing the utilization of the biomass matrix by systematically examining both the presence and concentrations of metabolites across different seasons and cultivation sites. This dual approach enhances our understanding of how environmental factors influence biomass quality, providing valuable insights for practical applications.

## 2. Experimental Design

### 2.1. Materials

The following materials were used:

- Fresh samples of the seaweed *Kappaphycus alvarezii*;
- Methanol (Dinâmica, Indaiatuba, São Paulo, Brazil, Cat. No.: P1000510180681);
- Acetic acid (Dinâmica, Indaiatuba, São Paulo, Brazil, Cat. No.: P10002100000);
- Distilled water;
- Folin–Ciocalteau reagent (Sigma-Aldrich, St. Louis, MO, USA, Cat. No.: TP0200);
- Whatman^®^ filter paper (Sigma-Aldrich, St. Louis, MO, USA, Cat. No.: WHA1004125);
- Sodium carbonate (Dinâmica, Indaiatuba, São Paulo, Brazil, Cat. No.: P10023403609);
- Sterile plastic containers;
- Glass cuvettes;
- Gallic acid (Sigma-Aldrich, St. Louis, MO, USA, Cat. No.: 27645);
- Aluminum chloride (Dinâmica, Indaiatuba, São Paulo, Brazil, Cat. No.: P10029400300);
- Quercetin (Sigma-Aldrich, St. Louis, MO, USA, Cat. No.: Q4951);
- 2,4,6-Tris(2-pyridyl)-s-triazine (Sigma-Aldrich, St. Louis, MO, USA, Cat. No.: T1253);
- Iron sulfate (Dinâmica, Indaiatuba, São Paulo, Brazil, Cat. No.: P1009600450027);
- 2,2-diphenyl-1-picryl-hydrazyl (Sigma-Aldrich, São Paulo, Brazil, Cat. No.: D9132);
- Acetone (Êxodo Cientifica, São Paulo, Brazil, Cat. No.: A09737RA);
- Tris hydrochloride (Sigma-Aldrich, St. Louis, MO, USA, Cat. No.: 108219);
- Extra virgin olive oil (Oli Ma, Arujá, São Paulo, Brazil, Lote: 584);
- Arabic gum (Synth, Diadema, São Paulo, Brazil, Cat. No.: G101206AG);
- Sodium hydroxide (Êxodo Cientifica, São Paulo, Brazil, Cat. No.: HS09639SO);
- Ethanol (Anidrol, Diadema, São Paulo, Brazil, Cat. No.: PAP.A-1213);
- Phenolphthalein (Dinâmica, Indaiatuba, São Paulo, Brazil, Cat. No.: P.10.0474.000.00.12);
- Casein (Synth, Diadema, São Paulo, Brazil, Cat. No.: C101406AF);
- Monobasic sodium phosphate (Synth, Diadema, São Paulo, Brazil, Cat. No.: F103401AG);
- Sodium phosphate dibasic (Synth, Diadema, São Paulo, Brazil, Cat. No.: F225401AG);
- Trichloroacetic acid (Dinâmica, Indaiatuba, São Paulo, Brazil, Cat. No.: P10002121900).

### 2.2. Equipment

The following equipment was used:

- Freeze-drying (Liobras, São Carlos, São Paulo, Brazil, Cat. No.: L101);
- Oven-drying process (DeLeo DL—AF, Porto Alegre, Brazil, Cat. No.: SE 42L);
- Ultrasound (Cristófoli, Campo Mourão, Paraná, Brazil, Cat. No.: USC100517);
- Centrifuge (Jouan BR4i, Saint-Herblain, France, Cat. No.: 1995/3777a);
- Vortex (Fisatom Mod. 772, São Paulo, Brazil, Cat. No.: 1563628);
- UV-vis spectrophotometer (BEL photonics^®^, São Paulo, Brazil, Cat. No.: 2000);
- Water bath (Solab, São Paulo, Brazil, Cat. No.: SL 150/10).

### 2.3. Solutions

The following solutions were used:

- Acidified methanol 80%: add 20 mL of acetic acid with 180 mL of distilled water to 800 mL of methanol;
- Buffered acetone: dissolve 4.8 g of Tris hydrochloride in 200 mL distilled water and add in 800 mL of acetone;
- Arabic gum solution, 7%: dissolve 7 g of Arabic gum in 100 mL phosphate buffer solution (0.1 M, pH 7.0);
- Acetone–ethanol: add 500 mL of acetone to 500 mL of ethanol;
- Sodium phosphate buffer: add about 400 mL monobasic sodium phosphate 0.1 M to about 500 mL sodium phosphate dibasic 0.1 M solution until pH 7 is obtained;
- Casein solution: dissolve 100 mg de casein in 100 mL distilled water.

## 3. Procedure

### 3.1. Sample Collection

**Figure 1:**
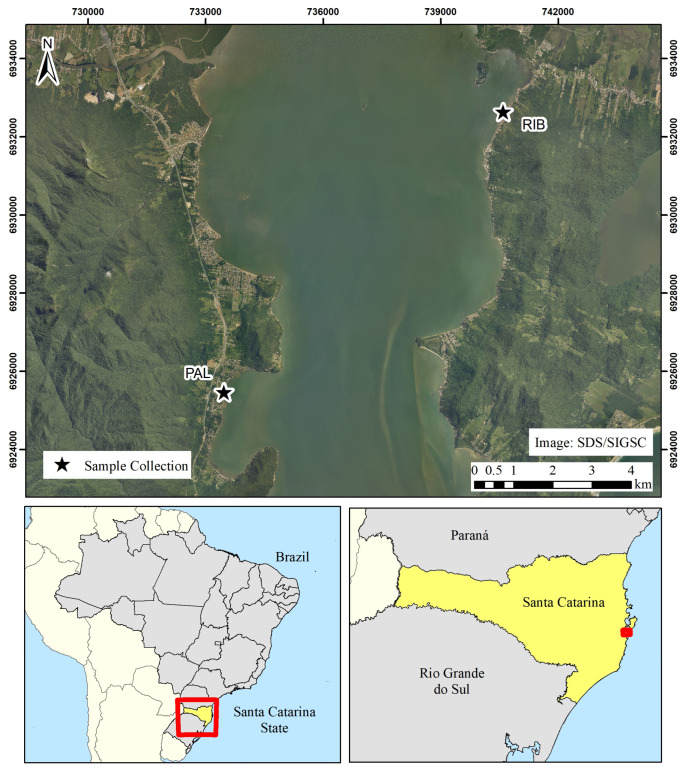
Details of the sites of collections of the macroalga *Kappaphycus alvarezii* in Florianópolis (RIB—Ribeirão da Ilha) and Palhoça (PAL), state of Santa Catarina, southern Brazil. Source: elaborated by the authors (2023).

Samples of *K. alvarezii* (lineage initially obtained from the State of São Paulo—Fisheries Institute, Ubatuba) were obtained from two marine farms located in the municipalities of Florianópolis (RIB—Ribeirão da Ilha, 27°42′32.7″ S, 48°33′35.5″ W) and Palhoça (PAL—27°46′29.9″ S, 48°37′50.7″ W), in the state of Santa Catarina, southern Brazil, in 2022. Santa Catarina state falls under the Cfa Köppen climate classification, with a subtropical climate with hot summers, mild winters, and evenly distributed rainfall throughout the year. Sampling was carried out seasonally as follows: two samples were collected in the late autumn (RIB-2 and PAL-2), four in the winter (early: RIB-3 and PAL-3; late: RIB-4 and PAL-4), four during the spring (early: RIB-5 and PAL-5; late: RIB-6 and PAL-6), and two in the early summer (RIB-7 and PAL-7), resulting in a total of twelve samples (Figure 1). To this end, 500 g samples of fresh *K. alvarezii* were randomly collected by the producers at the sampling locations and delivered to the laboratory on the same day for analysis. The producers were instructed to randomly select the algal samples from within the cultivation area to ensure representative sampling. Red and green thalli of *K. alvarezii* were sampled and mixed for the drying process.

The region experiences a subtropical climate, classified as humid oceanic without a distinct dry season and with hot summers (Cfa according to the Koppen system) [21]. This Cfa climate type is characterized by a maximum average temperature exceeding 22 °C and a minimum average between −3 and 18 °C. Rainfall is well distributed throughout the year [21], with an annual accumulated precipitation of 2016 mm recorded on Santa Catarina Island in 2023 [22]. During the period when the seaweed was collected, the observed average temperatures for the area referred to as PAL were 18.7 °C in the autumn, 18.5 °C in the winter, 21.7 °C in the spring, and 25.9 °C in the summer. For the RIB location, the recorded temperature averages were 18.1 °C (autumn), 17.9 °C (winter), 22.9 °C (spring), and 25.6 °C (summer). Regarding luminosity, the same conditions were recorded for both sites, with 11 h and 2 min of daylight in the autumn, 10 h and 51 min in the winter, 13 h and 11 min in the spring, and 13 h and 21 min in the summer [23].

### 3.2. Biomass Drying

The collected algal biomass was firstly freshwater-washed to remove salt, impurities, and encrusting organisms, followed by packaging in plastic bags and storage at −48 °C for subsequent drying by freeze-drying or oven-drying. Biomass samples (15 g, fresh material) were utilized in triplicate for each collection site and season (different parts of the seaweed), yielding an average dry weight of 2.5 g ± 0.7 g and 83.8% ± 1.7% moisture content for both drying methods investigated. Freeze-drying was carried out for 10 h (0.040 mbar vacuum, condensation chamber at −50 °C), while the oven-drying process (forced air convection drying oven with air circulation and renewal, with a fan turbine for air displacement and temperature controller) was carried out for seven consecutive days at ~60 °C, until constant biomass dry weight.

### 3.3. Total Phenolic Content

The methodology proposed by Singleton and Rossi [24] was used, with adaptations for the analysis of total phenolics. In short, 500 mg each of freeze-dried and oven-dried *K. alvarezii* samples was used, followed by adding 5 mL acidified methanol (80% methanol, 1% acetic acid, and 19% distilled water) as the extracting solvent. The samples were submitted to an ultrasonic bath (20 min) and centrifuged (5000 rpm, 10 min), and the supernatants were collected by filtration (filter paper 0.22 mm) under reduced pressure and stored in 10 mL amber glass flasks. The extraction process was repeated once again on precipitated material, and the supernatants were combined.

For the determination of the total phenolic content, a sample (0.5 mL) was added to 0.5 mL distilled water, 0.5 mL Folin–Ciocalteau reagent, and 2.5 mL sodium carbonate (20%, *w*/*v*). The test tubes were vortexed, and the reaction was allowed to proceed for 1 h. The absorbance reading was performed in glass cuvettes using a UV-vis spectrophotometer at 725 nm. For the calibration curve (y = 0.0282x, r^2^ = 0.9965), gallic acid was used as the analytical standard, at 0, 5, 15, 25, and 35 µg/mL concentrations.

### 3.4. Total Flavonoid Content

Total flavonoid content (TFC) was determined according to the methodology described by Awad et al. [25], with adaptations. Thus, 500 mg each of freeze-dried and oven-dried seaweed samples was transferred to 10 mL amber glass flasks, followed by the addition of 4 mL acidified methanol. After homogenization and ultrasonic bath treatment (30 min), 1 mL aluminum chloride (5%) was added, and a new homogenization was carried out. The obtained sample was kept in a dark environment for 30 min. Afterward, the sample was centrifuged (6000 rpm, 25 min; Jouan BR4) at 5 °C, and an aliquot (4 mL) was collected, followed by the addition of 1 mL aluminum chloride. The supernatant was removed, and the absorbance was read at 425 nm. The calibration curve (y = 0.013x, r^2^ = 0.9788) was performed using quercetin as the analytical standard at 20, 40, 60, 80, and 100 µg/mL concentration.

### 3.5. Antioxidant Activity

The antioxidant activity of the methanolic extract of *K. alvarezii* was determined using the ferric reducing antioxidant power (FRAP) [26] and 2,2-diphenyl-1-picryl-hydrazyl-hydrate (DPPH) [27] assays. For both analyses, 500 mg each of freeze-dried and oven-dried seaweed samples was used. For the FRAP assay, 10 mL acidified methanol was added to a 500 mg algal sample. After being submitted to an ultrasonic bath (10 min), the material was centrifuged (6000 rpm, 10 min at 5 °C). In test tubes, 900 µL FRAP reagent, 30 µL sample, and 90 µL distilled water were added. The blank was prepared in the same way, replacing the seaweed extract with 30 µL extracting solvent (acidified methanol). The absorbance reading was performed at 594 nm. The calibration curve was built with iron sulfate (FeSO_4_—y = 0.0007x, r^2^ = 0.9973) at 166.7, 333.3, 500.0, 666.7, 833.3, and 1000 mmol (*m*/*v*) concentration. The activity was expressed in mmol FeSO_4_ reduced per g fresh mass.

For the DPPH assay, biomass samples were weighed (500 mg) and transferred to falcon tubes (15 mL). Then, 10 mL methanol was added, followed by stirring (vortex) until homogenization and an ultrasonic bath treatment (15 min). The methanolic extract was recovered by centrifuging (3000 rpm, 10 min, 5 °C). In a test tube, 500 µL supernatant, 3 mL methanol, and 300 µL DPPH were added. The blank was prepared in the same way, by replacing the sample extract with 500 µL extracting solvent (acidified methanol). The reading of absorbance was performed at 517 nm. For the DPPH scavenging activity calculation, the following equation was used:

(1) $$DPPH\%=\frac{Blank\;absorbance-Sample\;absorbance}{Blank\;absorbance}\times 100$$

After obtaining the % reduced DPPH, a standard curve with Trolox at concentrations of 0.65, 1.31, 1.97, 2.63, 3.28, and 3.90 µg/mL (y = 23.606x, r^2^ = 0.9961) was built.

### 3.6. Total Carotenoids

For the analysis of total carotenoid content (TC), the methodology proposed by Sims and Gamon [28] was used. Oven-dried and freeze-dried *K. alvarezii* samples (100 mg) were each centrifuged (2000 rpm, 5 min) after the addition of 5 mL acetone, buffered with Tris hydrochloride (Tris-HCl), and vortex homogenization (5 min). After removing the supernatant, filtration was performed, and the absorbance of the organosolvent extract was measured in a glass cuvette at 663 nm (chlorophyll a), 647 nm (chlorophyll b), 537 nm (anthocyanin), and 470 nm (carotenoids). The calculation was performed according to the following equation:

(2) $$TC=\frac{A_{470}-\left(17.1\times \left(Chlorophylla+chlorophyllb\right)-9.479\times Anthocyanin\right)}{119.26}$$

### 3.7. Lipase and Protease Activities

The lipolytic activity of *K. alvarezii* samples was determined according to the method described by Okino-Delgado and Fleuri [29]. Briefly, dried samples (0.25 g) were mixed with 5 mL extra virgin olive oil and 7% Arabic gum solution (1:4, *v*/*v*) and 3 mL phosphate buffer solution (0.1 M, pH 7.0). Controls consisted of the same emulsion volume and phosphate buffer. The reaction mixture was incubated for 30 min at 40 °C in a thermostatic bath with agitation at 130 ppm (oscillations per minute). The reaction was stopped by the addition of an acetone–ethanol (1:1, *v*/*v*) solution. Fatty acids released by the hydrolysis of olive oil lipids were titrated with 0.05 M NaOH solution using phenolphthalein as an indicator. For the evaluation of the enzymatic activity, a lipolytic activity unit was defined as the amount of lipase able to release 1 μmol fatty acid per minute (U/g), under the described test conditions.

For the proteolytic activity, casein was used as a substrate, as described by Obata et al. [30], Rowley and Bull [31], and Ferracini-Santos and Sato [32], with modifications. The reaction mixture containing 1.5 mL casein solution (2% *w*/*v*), 1 mL sodium phosphate buffer (0.1 M, pH 7.0), and 50 mg sample was incubated in a water bath at 30 °C for 30 min. The reaction was stopped by adding 2 mL trichloroacetic acid (10% *w*/*v*), followed by centrifuging at 6000 rpm, for 15 min, at 4 °C. The absorbance was determined at 280 nm, and an activity unit was defined as the amount of protease able to increase the absorbance by one unit, under the test conditions, according to the following equation:

(3) $$\frac{U}{g}=\frac{\left(Sample\;absorbance-Blank\;absorbance)/0.1\right)}{0.05}$$

The results were expressed in U/g (stands for enzyme activity unit). The unit is defined by the number of moles of substrate transformed or product formed per gram (g) of enzyme extract used per minute of reaction, at standardized pH and temperature (i.e., assay conditions).

### 3.8. Statistical Analysis

All freeze-dried and oven-dried *K. alvarezii* samples were analyzed in triplicate (n = 3). Data were individually analyzed considering the two drying methods by ANOVA and Bonferroni correction (*p* ≤ 0.05) for the total contents of phenolic compounds, flavonoids, and carotenoids, besides antioxidant activity (i.e., DPPH and FRAP assays). For the lipase and protease activities, ANOVA was performed followed by Sidak’s multiple comparison test (*p* ≤ 0.05).

The Scott and Knott test [33] with a 5% error probability was applied for comparing means in order to analyze the collection sites, without considering the drying process, using scripts written in R language (v. 4.0.2). Subsequently, all variables were subjected to the principal component analysis (PCA) in order to verify the influence of drying methods between collection sites. PCA was calculated using the singular value decomposition (SVD) algorithm for matrix factorization. For the generation of PCAs, Unscrambler^®^ X (v. 10.4) statistical software was used. Linear discriminant analysis (LDA) was performed with support of the Past software (v. 4.03) as a classification model.

## 4. Results

Initially, when comparing the different drying protocols, no significant differences were observed (*t*-test, *p* = 0.678) in the moisture content and dry weight of *K. alvarezii* samples, regardless of season or collection site. Thus, it is possible to verify that both techniques enabled a drastic water reduction in the macroalgal biomass.

Freeze-dried *K. alvarezii* samples exhibited a higher total phenolic content (TPC) (39.23 to 127.74 mg GAE/100 g) than oven-dried ones (13.40 to 39.11 mg GAE/100 g), regardless of the collection site (Table 1). A comparative analysis applied to the contents of the secondary metabolites of interest for the freeze-dried samples from the two collection sites revealed a higher TPC in the early spring and late winter samples (PAL-5, RIB-5, and PAL-4, respectively) (Table 1).

For total flavonoid content (TFC), all samples, except RIB-3 (early winter) and PAL-4, differed statistically. Freeze-dried samples presented a wider range (4.10 to 15.62 mg QE/100 g) of TFC than the oven-dried ones (4.99 to 12.29 mg QE/100 g) (Table 1). Freeze-dried biomass revealed superior TFC amounts for the samples collected in the winter (RIB-3, RIB-4; early and late) and early spring (RIB-5).

In the FRAP analysis, all samples but RIB-6 and PAL-6 (late spring) and RIB-7 and PAL-7 (early summer) differed (*p* < 0.05) in their antioxidant activity between the two methods employed (Table 2). Most of the samples (i.e., 58%) showed a higher FRAP antioxidant activity when freeze-dried (RIB-4, RIB-5, RIB-7, PAL-2, PAL-3, PAL-4, and PAL-7—0.37 to 0.62 mmol/100 g) in comparison to oven-dried (0.28 to 0.70 mmol/100 g). Among the samples, freeze-dried PAL-4 (late winter) and oven-dried PAL-5 (early spring) presented the highest antioxidant activity (0.62 and 0.70 mmol/100 g, respectively), statistically differing from the others (Table 2).

All the samples analyzed differed in their antioxidant activity determined by the DPPH assay according to the drying methods, with superior values for the freeze-dried ones (6.12 to 8.91 mg/100 g) compared to the oven-dried samples (0.40 to 3.63 mg/100 g—Table 2). Interestingly, as noted in the FRAP assay, the early-spring-collected sample PAL-5 presented the highest DPPH activity for both the freeze-drying (8.91 mg/100 g) and oven-drying (3.63 mg/100 g) methods, statistically differing from other samples treated with the same drying method. This may be due to the accumulation of antioxidant compounds in early spring, possibly related to increased sunlight and temperature fluctuations.

The present study demonstrates higher DPPH antioxidant activity in freeze-dried samples compared to the FRAP method. However, no relationship was found between this variable and the locations of algal biomass collection, indicating wide variation between locations.

In a second approach, correlation analysis was applied to the TPC, TFC, FRAP, and DPPH dataset, considering the separation of samples according to their collection site (RIB and PAL) and drying methods (freeze-drying and oven-drying). The results are presented in Table 3. Significant correlations were found only between the TPC and DPPH variables for the freeze-dried PAL (r^2^ = 0.9886) and oven-dried (r^2^ = 0.7857) samples and when grouping RIB and PAL oven-dried samples (r^2^ = 0.7998).

Regarding the total carotenoid content (TC), firstly, there was a wide range of values in samples between the collection sites (i.e., RIB and PAL) and over the seasons studied, irrespective of the drying method adopted (Table 4). For this class of secondary metabolites, undoubtedly the freeze-dried protocol afforded better results, with superior amounts found in all samples, except for RIB-2 and PAL-6, which did not differ statistically (*p* < 0.05). Oven-drying proved ineffective for preserving carotenoids in *K. alvarezii* biomass, leading to quite important losses of these bioactive compounds in comparison to the lyophilization protocol. Interestingly, freeze-dried samples harvested over the winter and early spring presented the highest amounts of these pigments, regardless of the collection sites.

Surprisingly, lipase activity was not found in any studied samples, regardless of the method used for drying biomass.

In turn, protease activity was found in all examined samples, with values up to 886 times higher in oven-dried samples (RIB-2, Table 5). Most samples (75%) presented higher catalytic activity (*p* < 0.05) following oven-drying than freeze-drying, except RIB-7. Additionally, the highest catalytic activity detected in the oven-dried samples, i.e., RIB-2—1485.09 ± 26.84 U/g, was notoriously superior to that found in the freeze-dried one with the best performance, i.e., PAL-5, 579.25 U/g. Finally, as noted in the analysis of the secondary metabolites, protease activity does not seem to be related to the collection site of *K. alvarezii* and the seasonality, since a clear pattern of enzymatic activity was not detected with respect to these factors.

**Figure 2:**
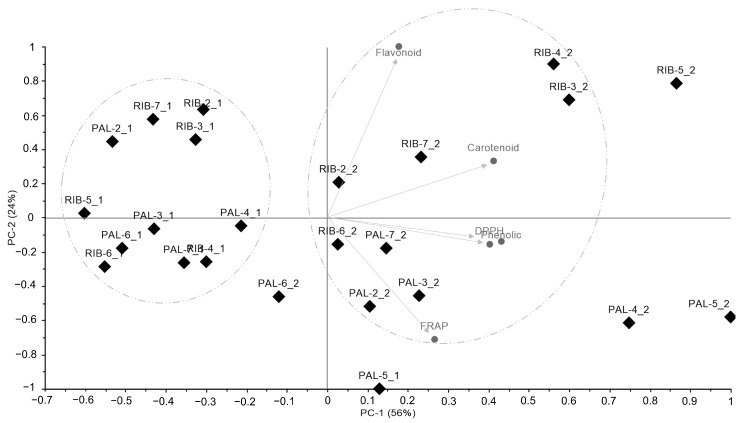
Score scatter plot and factor loadings of principal component analysis (PCA) of oven-dried and freeze-dried samples of *Kappaphycus alvarezii* cultivated in Ribeirão da Ilha (RIB, Florianópolis county) and Palhoça (PAL), southern Brazil. PAL-x_1 and RIB-x_1—oven-dried; PAL-x_2 and RIB-x_2—freeze-dried.

In a secondary approach to data analysis, multivariate statistical techniques were used, namely principal component analysis (PCA) and linear discriminant analysis (LDA), to verify the presence of clusters between the methodologies used. Protease activity was deemed irrelevant to the descriptive models built and thus excluded from the analysis. Further, PCA applied to the total phenolic, total flavonoid, FRAP, DPPH, and total carotenoid dataset revealed a total variance of 80%, with PC1 accounting for 56% and PC2 for 24% (Figure 2). Notably, the oven-dried samples (91.66%—n = 11) were distinctly separated along PC2 and were not associated with metabolites. This indicates that, in general, they exhibited lower concentrations of these compounds. Conversely, freeze-dried samples (clustered in PC1) were correlated with the computed variables. DPPH and phenolics showed a close association with carotenoids, while the flavonoid content exhibited an opposite relationship with FRAP. Regarding the harvest site and seasonality effects, no sample grouping was detected by PCA.

**Figure 3:**
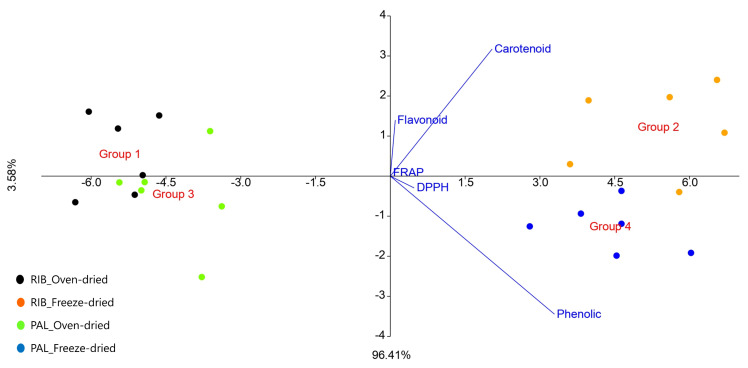
Linear discriminant analysis (LDA) of oven-dried and freeze-dried samples of *Kappaphycus alvarezii* cultivated in Ribeirão da Ilha in Florianópolis (RIB) and in Palhoça (PAL).

As a complementary model, LDA was applied, revealing that axes 1 and 2 accounted for 99.99% of the variance, with four distinct groupings identified, where RIB and PAL oven-dried samples (left quadrants) and RIB and freeze-dried PAL (right quadrants) appeared, as also seen via PCA (Figure 3). Total carotenoids and total flavonoids were the most relevant variables for the Group 2 samples (RIB) quadrant, while FRAP, DPPH, and total phenolics were determinants for the samples in the Group 4 (PAL) quadrant. In general, it is clear that the oven-dried samples, as seen in the PCA, were separated in the model, demonstrating that they have lower concentrations of compounds. Regarding the seasonality and cultivation sites of *K. alvarezii*, it can be observed that no distinct groups were formed. The differentiation occurred more prominently due to the difference between the oven-dried and freeze-dried methods.

## 5. Discussion

In analyzing the results obtained, we observe a quantitative variation in the analyzed compounds using different drying techniques, i.e., oven-drying and freeze-drying. However, an important factor to consider is the final moisture content after applying these techniques. In our study, we did not find significant statistical differences in the moisture content. Thus, our results differ from those reported by Nurshahida et al. [34], who employed the same drying methods but under different conditions. The authors used a 70 °C oven for overnight drying and −80 °C for freeze-drying over 72 h. Therefore, it can be inferred that changes in parameters can affect the moisture content results.

Barraca [35] describes that moisture contents above 35% in *K. alvarezii* can lead to degradation during storage and above 40% can result in the near-complete degradation of the carrageenan, which is the primary commercial application of this seaweed. Therefore, a 25% to 35% moisture content extends the shelf life of the seaweed for over 12 months [35]. However, further reducing the moisture content is essential to inhibit microbial growth, such as bacteria and fungi. For this, water activity should be considered and kept at 0.6 or less in seaweed [36]. In our study, we achieved a moisture reduction of 83.8% in the algal biomass by completely drying it for use in powder form, which allows for greater stability and shelf life.

Regarding phenolic compounds, it is important to highlight that their extraction from seaweed is highly attractive for applications in health-promoting products, and their preservation is essential during the initial phases of industrial processing [16]. Several phenolic compounds have been identified in *K. alvarezii*, including apigenin, cinnamic acid, chlorogenic acid, hispidulin, isoorientin, isoquercitrin, kaempferol, methoxyphenylacetic acid, naringenin, salicylic acid, scopoletin, rutin, and xanthohumol [6].

When analyzing the type of drying for the results obtained for total phenolic content and total flavonoid content, it was found that, in both cases, higher levels and/or a broader range were achieved when using freeze-drying. This factor arises from the ability to better preserve the biochemical properties of the compounds, minimizing the degradation that can occur at higher temperatures and maintaining the structural integrity of the molecules [37]. This result is supported by studies conducted by Paga et al. [38], which investigated the influence of drying methods on *Sargassum* sp., and by Ullah et al. [39], who evaluated the chemical constituents of *Gracilariopsis longissimi*.

When analyzing secondary variables, e.g., collection location and seasonality, Araújo et al. [40] found contents between 40.80 and 58.49 mg GAE 100 g^−1^ in cultivated *K. alvarezii* biomass from southeastern Brazil, while Nurshahida et al. [41] reported a TPC at 1917 mg GAE 100 g^−1^ in samples produced in Malaysia. As shown herein, the comparison of contents of *K. alvarezii*’s phenolic compounds in samples harvested at two collection sites (PAL and RIB) situated in the same bay and 10 km away from each other (Figure 1) revealed a high variability within the sampling sites, without a clear difference between the production areas. These findings highlight the impact of production and harvest seasons on the metabolic profile of *K. alvarezii* cultured in southern Brazil. Seasonality significantly influences the phenolic content in algal biomass, particularly during mild temperatures in winter and early spring, especially in freeze-dried samples (Table 1). This aligns with previous results by Araújo et al. [42], which noted higher phenolic levels in green and red phenotypes of *K. alvarezii* in southeastern Brazil during the summer and autumn. The increase in phenolic compounds during these seasons may result from greater sunlight exposure and temperature, stimulating secondary metabolite production as a protective response to environmental stressors [43,44]. Therefore, the harvest season can affect the bioactive compound content, influencing the quality of the raw material for industrial applications.

Regarding the total flavonoid content, samples exhibited high variability across seasons and collection sites. Our findings indicate that *K. alvarezii* cultured under mild temperatures (winter and early spring) synthesizes and accumulates higher TFC values, particularly in freeze-dried samples. Interestingly, similar results were observed for TPC in samples collected in early spring. In contrast, oven-dried samples showed greater variability in secondary metabolite concentrations, lacking a clear seasonal pattern. Previous studies suggested that the TFC in *K. alvarezii* may be higher in oven-dried samples. Ling et al. [45] reported that oven-dried samples at 40 °C and 80 °C had flavonoid contents of 25.67 and 23.67 mg/100 g, respectively, compared to 12.33 mg/100 g in freeze-dried biomass. Charles et al. [46] also found a higher flavonoid content in oven-dried *K. alvarezii* biomass (0.13 mg/g) compared to freeze-dried samples (0.09 mg/g). For red algae, the optimal extraction temperature for bioactive compounds like flavonoids is typically between 40 °C and 60 °C, preserving their integrity and preventing degradation at higher temperatures [47,48].

Conversely, the drying method can interfere with the phenolic content in seaweed. For example, the TPC can be reduced in oven-dried and sun-dried samples compared to in freeze-dried samples, an effect attributed to the high processing temperatures, which can result in the rapid oxidation of those secondary metabolites [15,49]. Thus, oven-drying, commonly used to reduce operating processing costs, generally leads to phenolic content reduction [50]. Freeze-drying, based on the phenomenon of water sublimation from a previously frozen sample under reduced pressure, can better preserve product quality attributes such as nutrients, color, and flavors, although this method is generally more expensive compared to other drying techniques. In fact, lyophilization guarantees minimal thermal damage and good preservation of flavors, nutrients, and bioactivities, besides a low final moisture content in the product [50]. Low temperatures might promote the greater stability of phenolics for longer periods, which may be related to the inhibition of phenol oxidase activity, thus reducing oxidation and, consequently, condensation and degradation. These results were confirmed by studies carried out with other seaweed species such as *Padina pavonica* [51], *Cladophora glomerata* [52], *Sargassum fusiforme* [53], and *Cytosphora* sp. [19].

Regarding antioxidant capacity, the DPPH assay consistently exhibited higher activity compared to the FRAP method. In particular, freeze-dried samples demonstrated a greater antioxidant capacity than their oven-dried counterparts in the DPPH assay. These findings highlight the importance of both the choice of antioxidant activity method and the drying process, which significantly influence the bioavailability of antioxidants in seaweed. Antioxidant compounds, including polyphenols, carotenoids, and sulfated polysaccharides, protect against oxidative stress by scavenging reactive oxygen species (ROS) and modulating cellular signaling pathways [54,55,56]. Studies have shown that freeze-drying effectively preserves higher total amino acid levels and physico-chemical properties, thereby enhancing the potential of seaweed as a food ingredient [57]. Furthermore, freeze-drying has been associated with increased concentrations of metabolites that may enhance the bioavailability of antioxidants in these organisms [58]. In contrast, the oven-drying process can degrade thermolabile compounds, negatively affecting antioxidant levels and their overall bioavailability [59].

Previous reports indicate that *K. alvarezii* exhibits an excellent radical scavenging effect both by FRAP and DPPH assays [45,60,61]. In fact, the antioxidant activity determined by the FRAP assay reported in the present study was higher (0.28–0.70 mmol/100 g) than that recorded in *K. alvarezii* cultivated in two sites in Malaysia, e.g., 0.010 mmol/100 g (Langkawi, Kedah) and 0.017 mmol/100 g (Semporna, Sabah) [61]. Lower values have also been found for the same species cultivated in Indonesia, where FRAP activity varying from 0.45 to 0.97 mM FeSO_4_ g^−1^ (0.045 to 0.097 mmol/100 g) was detected among sun-dried, oven-dried, vacuum-assisted drying, and freeze-dried samples [46]. Regarding the DPPH test, Araújo et al. [40] observed that *K. alvarezii* cultivated in southeastern Brazil exhibited values ranging from 7.15% to 31.21%, which are similar to the observations found herein. Taking together, these results indicate that large variations in the antioxidant activity of *K. alvarezii* have been detected in samples originating from climatically distinct cultivation regions worldwide. This difference may be due to seasonal variations, as our study was conducted in an environment with more distinct seasonal changes, which may promote a higher accumulation of antioxidant compounds compared to more constant climates such as that in Malaysia.

When analyzing the correlation between polyphenol levels and antioxidant activity, a low correlation was observed for most of the studied samples. This suggests that compounds other than phenolics, such as the polysaccharide carrageenan, pigments (chlorophylls, carotenoids, and phycobiliproteins), proteins, and peptides [62], may be responsible for the antioxidant activity of the *K. alvarezii* samples investigated.

Regarding carotenoids, freeze-drying proved to be a more efficient method for their preservation in the algal biomass, exhibiting lower pigment degradation. This can be attributed to the high susceptibility of carotenoids to heat- and oxidation-related decay [63], similar to polyphenols. Studies have reported higher contents of total carotenoids (0.26 to 0.52 mg g^−1^ [64] and 0.38 to 0.53 mg g^−1^ [62]) than those found herein. These discrepant values with respect to the present work can be ascribed to the fact that the above-mentioned authors carried out measurements on fresh algal biomass, differently from those performed in this study.

In addition to analyzing secondary metabolite contents, we measured lipase and protease activities in the *K. alvarezii* biomass. Lipases catalyze the hydrolysis of triglycerides, oils, and fats, while proteases hydrolyze peptide bonds [65]. Both enzymes are widely used in industries such as food, beverages, detergents, animal feed, personal care, cosmetics, nutraceuticals, and textiles [66,67,68]. Marine organisms, including *K. alvarezii*, are gaining attention as enzyme sources due to their stability and activity compared to plant- and animal-derived enzymes [69]. Thus, this analysis aims to highlight the biotechnological potential of *K. alvarezii* as an enzyme source for industrial applications.

Lipase activity was not found in our study, and this result may be related to the low lipid concentration (0.36–1.95%) of *K. alvarezii*, as previously reported [70,71]. However, results were obtained regarding protease. *K. alvarezii*, especially oven-dried biomass, showed relevant values of proteolytic activity, which may be of great interest for industry. It is possible that oven-drying protocols may increase the availability of proteases and their activity due to efficient biomass destruction at high temperatures, 50–60 °C [72,73]. In comparison to other macroalgal species, *K. alvarezii* presents superior protease activity compared to *Ulva rigida*, *Codium decorticatum*, *Stypocaulon scoparium*, *Dictyota dichtoma*, *Pterocladiella capillacea*, and *Gracilaria* sp. [74].

In general, it can be concluded that the freeze-drying process more effectively preserves the important bioactive compounds of *K. alvarezii* biomass in comparison to the oven-drying technique. However, depending on the specific application, oven-drying may be preferable for certain industrial applications of the algal biomass, making it suitable for various uses. According to Stratta et al. [75], a comprehensive economic analysis of the freeze-drying process suggests that the costs involved may not be as prohibitive as the initial investment required for setting up operations. However, in adopting industrial drying processes based on lyophilization, it is essential to consider whether significant advantages in preserving compounds are relevant or if a risk exists of compromising quality when opting for alternative and lower-cost drying methods, such as oven-drying. Additionally, the selection of industrial dryers must consider other factors like energy consumption and environmental impact, particularly when prioritizing sustainability and the utilization of natural products [76].

This consideration is particularly important due to the necessity of cost-effectiveness in industrial applications involving the seaweed species investigated herein. In a previous study conducted by our research group [77], diverse applications of *K. alvarezii* biomass have been described, such as cosmetics, animal nutrition, human food, health/medicine, agriculture, and general industry, in connection with the quality of the seaweed raw material used. This underscores the significance of investigating the impact of the drying process on the quality of the *K. alvarezii* produced, particularly in regions where its cultivation is still in nascent stages, as is the case in Brazil.

## 6. Conclusions

Comparison between freeze-drying and oven-drying methods for processing *Kappaphycus alvarezii* biomass, as reported here, indicates that freeze-dried samples generally exhibit higher compound concentrations. These results underscore the significance of the drying process in preserving bioactive compounds in seaweed biomass, enabling the selection of the most appropriate method for specific industrial applications. Freeze-drying is recommended for industries such as pharmaceuticals and cosmetics, where the aim is to obtain biomass with a higher compound richness or greater stability.

Further investigations are warranted to assess the impact of drying methods on other bioactive compounds present in *K. alvarezii* biomass, e.g., amino acids, biogenic amines, and phytohormones. Additionally, exploring the influence of geographic origin and seasonality may help optimize cultivation practices across different regions and seasons of the year.

## Figures and Tables

**Table 1:** Total phenolic content (TPC, mg GAE/100 g) and total flavonoid content (TFC, mg QE/100 g) of freeze-dried and oven-dried *Kappaphycus alvarezii* biomass.

Sample	Phenolics		Flavonoids	
	Freeze-Dried	Oven-Dried	Freeze-Dried	Oven-Dried
RIB-2	59.54 ± 0.39 ^e^	18.44 ± 0.11 ^g^	9.05 ± 0.16 ^e^	12.29 ± 0.17 ^a^
RIB-3	67.42 ± 0.19 ^d^	17.56 ± 0.46 ^h^	10.92 ± 0.21 ^c^	11.02 ± 1.14 ^b ns*^
RIB-4	49.05 ± 0.96 ^f^	22.52 ± 0.41 ^e^	15.62 ± 0.12 ^a^	4.99 ± 0.29 ^e^
RIB-5	98.51 ± 0.37 ^c^	21.20 ± 0.28 ^f^	13.23 ± 0.06 ^b^	5.86 ± 0.30 ^d^
RIB-6	44.30 ± 0.16 ^h^	13.40 ± 0.44 ^j^	6.75 ± 0.10 ^h^	5.05 ± 0.04 ^e^
RIB-7	44.45 ± 0.14 ^h^	14.51 ± 0.50 ^i^	9.31 ± 0.05 ^d^	10.51 ± 0.08 ^b^
PAL-2	39.23 ± 1.27 ^j^	23.04 ± 0.39 ^d^	6.37 ± 0.04 ^i^	7.81 ± 0.07 ^c^
PAL-3	41.82 ± 0.29 ^i^	23.52 ± 0.04 ^d^	5.58 ± 0.06 ^j^	6.45 ± 0.41 ^d^
PAL-4	105.57 ± 0.31 ^b^	29.68 ± 0.17 ^c^	7.77 ± 0.06 ^f^	8.37 ± 0.08 ^c ns*^
PAL-5	127.74 ± 0.41 ^a^	39.11 ± 0.23 ^a^	7.05 ± 0.12 ^g^	5.45 ± 0.38 ^e^
PAL-6	43.99 ± 0.73 ^h^	14.90 ± 0.19 ^i^	4.10 ± 0.08 ^k^	5.31 ± 0.09 ^e^
PAL-7	45.90 ± 0.23 ^g^	31.97 ± 0.93 ^b^	6.79 ± 0.07 ^h^	5.90 ± 0.15 ^d^

RIB-2 and PAL-2: late autumn; RIB-3 and PAL-3: early winter; RIB-4 and PAL-4: late winter; RIB-5 and PAL-5: early spring; RIB-6 and PAL-6: late spring; RIB-7 and PAL-7: early summer. Means followed by the same letter in the column do not differ statistically by the Scott–Knott test (*p* ≤ 0.05). ns* = not significant for analysis between types of drying method within the same variable (TPC and TP—line) by Bonferroni correction (*p* ≤ 0.05).

**Table 2:** Antioxidant activity by FRAP (mmol/100 g) and DPPH reducing capacity (mg/100 g and %) of *Kappaphycus alvarezii* macroalgal biomass after freeze-drying and oven-drying.

Sample	FRAP		DPPH	
	Freeze-Dried	Oven-Dried	Freeze-Dried	Oven-Dried
RIB-2	0.37 ± 0.01 ^h^	0.43 ± 0.01 ^d^	6.12 ± 0.35 ^g^ (38.69)	0.40 ± 0.05 ^f^ (7.29)
RIB-3	0.40 ± 0.01 ^g^	0.43 ± 0.01 ^d^	6.75 ± 0.04 ^f^ (42.42)	0.80 ± 0.03 ^e^ (11.98)
RIB-4	0.48 ± 0.01 ^e^	0.45 ± 0.02 ^c^	7.99 ± 0.25 ^c^ (49.77)	1.06 ± 0.06 ^d^ (15.04)
RIB-5	0.45 ± 0.01 ^f^	0.33 ± 0.02 ^g^	6.24 ± 0.07 ^g^ (39.46)	0.71 ± 0.01 ^e^ (11.00)
RIB-6	0.41 ± 0.00 ^g^	0.42 ± 0.01 ^d ns*^	7.58 ± 0.07 ^d^ (47.37)	0.41 ± 0.07 ^f^ (7.42)
RIB-7	0.38 ± 0.00 ^h^	0.36 ± 0.01 ^f ns*^	7.64 ± 0.14 ^d^ (47.73)	0.87 ± 0.02 ^e^ (12.83)
PAL-2	0.54 ± 0.02 ^c^	0.28 ± 0.01 ^h^	6.56 ± 0.08 ^f^ (41.33)	1.07 ± 0.26 ^d^ (15.17)
PAL-3	0.52 ± 0.00 ^d^	0.41 ± 0.01 ^e^	6.83 ± 0.02 ^e^ (42.92)	0.81 ± 0.15 ^e^ (12.17)
PAL-4	0.62 ± 0.01 ^a^	0.49 ± 0.00 ^b^	8.25 ± 0.06 ^b^ (51.32)	1.57 ± 0.09 ^c^ (21.16)
PAL-5	0.60 ± 0.01 ^b^	0.70 ± 0.01 ^a^	8.91 ± 0.10 ^a^ (55.18)	3.63 ± 0.12 ^a^ (45.38)
PAL-6	0.39 ± 0.01 ^g^	0.40 ± 0.00 ^e ns*^	6.90 ± 0.08 ^e^ (43.32)	1.01 ± 0.08 ^d^ (14.52)
PAL-7	0.45 ± 0.01 ^f^	0.43 ± 0.01 ^d ns*^	6.99 ± 0.06 ^e^ (43.87)	2.30 ± 0.15 ^b^ (29.69)

RIB-2 and PAL-2: late autumn; RIB-3 and PAL-3: early winter; RIB-4 and PAL-4: late winter; RIB-5 and PAL-5: early spring; RIB-6 and PAL-6: late spring; RIB-7 and PAL-7: early summer. Means followed by the same letter in the column do not differ statistically by the Scott–Knott test (*p* ≤ 0.05). ns* = not significant for analysis between types of drying method within the same variable (FRAP and DPPH—line) by Bonferroni correction (*p* ≤ 0.05).

**Table 3:** Correlation analysis of the antioxidant capacity and total phenolic and flavonoid contents of freeze-dried and oven-dried *Kappaphycus alvarezii* samples cultivated in Florianópolis (RIB) and Palhoça (PAL), southern Brazil.

Sample	Drying Method	Phenolic Content	Correlation Coefficients	
			FRAP	DPPH
RIB	Freeze-dried	TPC	y = 0.0006x + 0.3782 r^2^ = 0.0867	y = −0.0283x + 8.7698 r^2^ = 0.5494
		TFC	y = 0.0111x + 0.2951 r^2^ = 0.677	y = 0.0226x + 6.8088 r^2^ = 0.0083
	Oven-dried	TPC	y = 0.0004x + 0.3956 r^2^ = 0.0008	y = 0.0346x + 0.0871 r^2^ = 0.2274
		TFC	y = 0.0004x + 0.3987 r^2^ = 0.001	y = −0.0149x + 0.8307 r^2^ = 0.0367
PAL	Freeze-dried	TPC	y = 0.0017x + 0.4079 r^2^ = 0.5512	y = 0.0241x + 5.7845 r^2^ = 0.9886
		TFC	y = 0.0551x + 0.1755 r^2^ = 0.651	y = 0.4371x + 4.665 r^2^ = 0.3578
	Oven-dried	TPC	y = 0.0128x + 0.1052 r^2^ = 0.5708	y = 0.113x + 1.3253 r^2^ = 0.7857
		TFC	y = −0.0432x +0.7332 r^2^ = 0.1497	y = −0.3212x + 3.8339 r^2^ = 0.1458
RIB + PAL	Freeze-dried	TPC	y = 0.0016x + 0.363 r^2^ = 0.3259	y = 0.0131x + 6.3952 r^2^ = 0.2112
		TFC	y = −0.0035x + 0.4979 r^2^ = 0.0178	y = −0.0004x + 7.2343 r^2^ = 2 x 10^-6^
	Oven-dried	TPC	y = 0.0088x + 0.229 r^2^ = 0.4331	y = 0.1052x + 1.1477 r^2^ = 0.7998
		TFC	y = −0.0079x + 0.4852 r^2^ = 0.0385	y = −0.1205x + 2.1126 r^2^ = 0.1146

TPC: total phenolic content; TFC: total flavonoid content.

**Table 4:** Total carotenoid concentration (µg/g) of *Kappaphycus alvarezii* biomass after freeze-drying and oven-drying.

Sample	Carotenoids	
	Freeze-Dried	Oven-Dried
RIB-2	13.01 ± 1.80 ^g^	14.37 ± 0.40 ^c ns*^
RIB-3	72.76 ± 0.17 ^a^	13.15 ± 0.70 ^d^
RIB-4	47.02 ± 0.34 ^c^	27.19 ± 0.64 ^a^
RIB-5	75.33 ± 10.22 ^a^	2.19 ± 0.14 ^h^
RIB-6	15.76 ± 2.22 ^f^	6.05 ± 0.12 ^g^
RIB-7	39.35 ± 2.12 ^d^	11.42 ± 0.80 ^e^
PAL-2	19.31 ± 2.68 ^f^	9.89 ± 1.30 ^f^
PAL-3	37.96 ± 1.27 ^d^	11.18 ± 0.03 ^e^
PAL-4	34.73 ± 0.87 ^d^	14.79 ± 0.58 ^c^
PAL-5	54.37 ± 0.55 ^b^	21.47 ± 1.42 ^b^
PAL-6	10.27 ± 1.99 ^g^	9.69 ± 0.63 ^f ns*^
PAL-7	28.93 ± 1.27 ^e^	4.92 ± 0.58 ^g^

RIB-2 and PAL-2: late autumn; RIB-3 and PAL-3: early winter; RIB-4 and PAL-4: late winter; RIB-5 and PAL-5: early spring; RIB-6 and PAL-6: late spring; RIB-7 and PAL-7: early summer. Means followed by the same letter in the column do not differ statistically by the Scott–Knott test (*p* ≤ 0.05). ns* = not significant for analysis between types of drying method within the same variable.

**Table 5:** Protease activity (U/g) of *Kappaphycus alvarezii* biomass after freeze-drying and oven-drying.

Sample	Protease Activity	
	Freeze-Dried	Oven-Dried
RIB-2	150.47 ± 1.43 ^g^	1485.09 ± 26.84 ^a^
RIB-3	204.01 ± 8.47 ^e^	744.46 ± 53.19 ^b^
RIB-4	308.18 ± 10.53 ^c^	431.70 ± 13.53 ^e^
RIB-5	174.40 ± 1.08 ^f^	298.50 ± 6.67 ^g^
RIB-6	75.98 ± 2.31 ^i^	221.71 ± 28.27 ^h^
RIB-7	92.50 ± 0.62 ^h^	119.50 ± 9.50 i ^ns*^
PAL-2	155.34 ± 11.01 ^g^	393.82 ± 2.60 ^f^
PAL-3	245.65 ± 7.23 ^d^	654.07 ± 13.92 ^c^
PAL-4	466.86 ± 8.44 ^b^	366.77 ± 1.23 ^f^
PAL-5	579.25 ± 0.45 ^a^	503.97 ± 22.82 ^d^
PAL-6	90.00 ± 8.41 ^h^	131.59 ± 6.59 ^i^
PAL-7	474.62 ± 16.36 ^b^	315.29 ± 1.80 ^g^

RIB-2 and PAL-2: late autumn; RIB-3 and PAL-3: early winter; RIB-4 and PAL-4: late winter; RIB-5 and PAL-5: early spring; RIB-6 and PAL-6: late spring; RIB-7 and PAL-7: early summer. Means followed by the same letter in the column do not differ statistically by the Scott–Knott test (*p* ≤ 0.05). ns* = not significant for analysis between types of drying method within the same variable.

## Data Availability

The data in this manuscript are available and can be provided to researchers upon request. Interested parties are invited to contact the authors of this manuscript for access to the data.
